# Research on Detection and Early Warning Mechanism of Emergency Public Health Medical Education System Based on Internet of Things Technology

**DOI:** 10.1155/2022/3008206

**Published:** 2022-06-24

**Authors:** Lu Fang, Caixia An, Bin Yi

**Affiliations:** Gansu Provincial Maternity and Chlid-Care Hospital, Lanzhou 730050, China

## Abstract

Sudden public health and medical education events have tested the stability of society to a great extent. The government need to strengthen capacity building, make use of system dynamic supervision, warn public health events in advance, and minimize the impact scope and related harmfulness of events. This not only facilitates the rapid mobilization of resources by the later government but also facilitates the comprehensive and detailed deployment and arrangement of decision-makers. As we all know, the Internet of Things is used by all walks of life because of its outstanding advantages of low power consumption, low cost, and wide range. Therefore, this article takes the Internet of Things as the technical basis of the system. According to the actual demand and resource design, it includes two system functions: detection and early warning. The results show that: (1) considering the practical principle, the evaluation system interface found that the scores of font size and color style are all below 80%, which need to be optimized and adjusted; the overall interface basically meets the needs of users. (2) The throughput of the three methods is different. The CoAP-E has superior throughput. (3) With the increase in packet loss rate, the request success rate of the CoAP method decreases in a “drop” manner. The CoAP-E method in this article has the best performance. (4) When the packet loss rate is 25%, the network adaptability of this method is the strongest, and the retransmission rate is less than 18%; the CoAP method is as high as 35%. (5) When the number of concurrent requests is less than 2500, there is no obvious difference between the three methods; the optimal performance of the dynamic load balancing method is 10.1 s. (6) The system comprehensively considers various factors of five site selections. The highest comprehensive score of Final Site, 5 is 8.7, which can be used as the resettlement place of emergency rescue facilities. This article starts from the characteristics and needs of public emergencies, and the final set of the system runs well. It can quickly reflect public health emergencies and medical education events. Use the most effective system functions for risk control, and maximize the analysis, organization, and coordination of events. The follow-up optimization of system details needs to be studied.

## 1. Introduction

It is necessary and important to study public health care and educational events in emergencies. This process involves many rules, procedures, and methods. There are many basic theories to study this aspect, but the design and implementation of specific systems need to be comprehensively analyzed and integrated. The existing detection and early warning processing system in China have not yet defined the specific responsibilities, the functions are not perfect, and there are many detections and early warning problems. For example, the current systematic detection of public health emergencies and medical education in China is slow and complicated. In addition, the current system does not use new computer technology, the structure is old and the operation is backward, and it usually consumes a lot of funds and human resources, so it cannot respond to the demand quickly and timely. As for the early warning mechanism, first, the boundaries of authority are unclear, the responsibilities are vague, and there is no specific detail of the division problem. Second, the system's early warning mechanism design is unreasonable and unscientific, which cannot perfectly coordinate the conflict between public and personal interests. The reserve of professional knowledge talents for early warning is insufficient, the early warning mechanism lacks corresponding laws and regulations, and there are problems with the system and mechanism. The system is assisted by GIS, load balancing, big data, and other technologies. The system designed in this article can properly reflect the situation of interest protection and power coordination when people and government encounter unexpected situations. The following documents can provide a valuable reference for the construction of emergency detection and early warning system designed in this article.

According to the need for emergency communication support in typical scenarios, an emergency communication system is constructed [1]. According to the existing emergency support communication system, a 5G + air-space integrated emergency support system is proposed [2]. In view of the application of high-speed rail, we should further improve the transportation support level of emergency materials and study the emergency support system for public health emergencies [3]. Focusing on the logistics support work carried out by emergency command organizations and executive agencies, this article studies the emergency support system of agency affairs [4]. This article systematically analyzes the emergency guarantee system of public health safety of urban water system and puts forward emergency capability, technical requirements, overall emergency strategy, and “5 + 3” emergency plan [5]. Based on the INSARAG system, a general module of emergency rescue drug support was established [6] to strengthen the construction and management of the emergency system [7]; improve the construction of emergency plans and explore the construction mode of emergency support mode of clinical medical engineering in medical rescue [8]; discuss the management level and quality of emergency materials logistics support [9]; construct and evaluate the emergency management system of general wards during novel coronavirus pneumonia [10]; and improve the strategic function of the human information system and discuss the role of hospitals in preventing and treating public health emergencies [11]. In view of public health emergencies, this article takes the epidemic situation in novel coronavirus in 2019 as an example to [12] support the emergency response mechanism of the public health safety system with “big data + grid” [13] and establish a remote intelligent prevention and control diagnosis platform based on artificial intelligence under sudden epidemic situations [14]. From the global emergencies, aiming at the oral medical and public health system, we should make differentiated protection and emergency plans at different levels [15]. To sum up, we learn from each other's strengths and selectively select valuable and advantageous research results as the theoretical basis of this article. This can effectively help us understand the problem from the essence.

## 2. Theoretical Basis

### 2.1. Internet of Things Technology

IoT [16], one of its important infrastructures is sensors. The agreed transmission protocol mainly completes the communication between people and things and effectively manages and monitors the connection between goods and people. Therefore, it is closely related to cloud computing, big data, communication, embedded hardware, and other technologies. It has more application prospects in many fields such as smart cities, artificial intelligence, and home medicine, serving human society and bringing excellent experience effects to users.

IoT is called the Internet of Things technology, which means “everything is connected to each other into a network (Internet).” Through the sensor, a large amount of data collection can be carried out effectively. This technology shows the advantages of low power consumption, low cost, wide-coverage, and a large number of connections. The most commonly used system architecture on the Internet of Things technology is the transmission system based on NB-IoT. Our platform unifies the data of all devices that can be connected to the Internet of Things.

**Figure 1:**
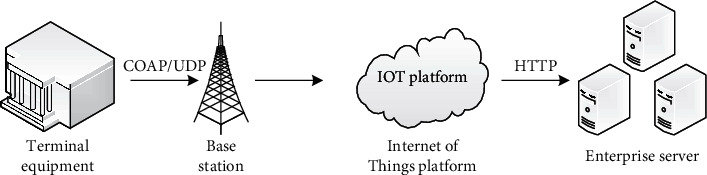
Internet of things transmission architecture.

The technical architecture represented by it combines terminal equipment, NB-IoT controller, and sensor settings into a community. It is transmitted to the base station by COAP/UDP protocol, and the operator reports the data collected by the sensor to the IoT platform, that is, the Internet of Things platform. At the same time, the platform can also use the HTTP protocol to take the enterprise server as a third-party application system, and open the interface, which is convenient for relevant professionals to manage enterprise equipment. The service performance transmitted by this architecture is extremely superior (Figure 1).

### 2.2. Public Health Emergencies

#### 2.2.1. Demarcation of Event Boundaries

Emergencies [17] generally refer to sudden events in our ordinary daily life. Such events are often unexpected or unexpected. From a specific point of view, emergencies usually refer to public emergencies. Up to now, there is no clear, official, and unified definition of public emergencies. But we collect many experts and scholars of the more representative explanations and made a certain summary. We can analyze and compare the characteristics of several events, to have a deeper understanding of the boundaries of public emergencies (Table 1).

#### 2.2.2. Public Health Emergencies

There are various public emergencies in the world, especially in health, medical treatment, and education, such as biological, chemical, major infectious diseases, campus gun battles, and other events that belong to the division scope of this event. Because of its sudden occurrence, people are caught off guard. It is easy to cause or likely to cause widespread panic. When such an incident occurs, it is important for various departments and organizations to take various measures to deal with it as quickly as possible in the shortest reflection time to minimize the loss of personnel and property to a certain extent. Specific event classification and grading are shown in Table 2.

### 2.3. Facility Location

After the occurrence of public emergencies, to solve the problem most quickly, we need to choose the best address as the dispatching center according to the central position of the event to meet the needs of various job transfers. The aim is to shorten the distance from the event center under the condition of meeting the decision conditions. For traditional site selection, there is continuous and discrete site selection.

#### 2.3.1. Continuous Site Selection

(1) $$\begin{array}{c} \left(x,x\right)\in R*R, \end{array}$$

(2) $$\begin{array}{c} \min\left(x,y\right)\sum_{i\in I}w_{i}d_{i}\left(x,y\right), \end{array}$$

(3) $$\begin{array}{c} \left(x,y\right)\sqrt{{\left(x-a_{i}\right)}^{2}+{\left(y-b_{i}\right)}^{2}}. \end{array}$$

#### 2.3.2. Discrete Site Selection

(4) $$\begin{array}{c} \bar{x}=\frac{x_{1}f_{1}+x_{2}f_{2}+x_{3}f_{3}+\ldots x_{k}f_{k}}{k}. \end{array}$$

Among them, *a*_*i*_ and *b*_*i*_ refer to each demand point; *w*_*i*_*d*_*i*_(*x*, *y*) represents the weighted distance from the event center; *w*_*i*_ indicates that the weight corresponding to the demand point is small; *i* refers to the standard set.

The model established in this article is as follows:

(5) $$\begin{array}{c} \text{Min}L, \end{array}$$

(6) $$\begin{array}{c} ST: \end{array}$$

(7) $$\begin{array}{c} \sum_{j\in I}z_{ij}p_{jk}=Q_{i}\forall i\in I, \end{array}$$

(8) $$\begin{array}{c} z_{ij}\leq x_{i}\forall i\in I,j\in J, \end{array}$$

(9) $$\begin{array}{c} L\geq \frac{\sum_{j\in J}\beta_{ik}e_{ik}M_{i}{\text{d}}_{ij}z_{ij}}{Q_{i}}\forall i\in I,k\in K, \end{array}$$

(10) $$\begin{array}{c} x_{j},z_{ij}=\left\{0,1\right\}\forall i\in I,j\in J. \end{array}$$

If we need two emergency facilities, then we need to modify two functions, as shown:

(11) $$\begin{array}{c} MinL_{1}+L_{2}, \end{array}$$

(12) $$\begin{array}{c} L_{r}\geq \frac{\sum_{j\in J}\beta_{ik}e_{ik}M_{i}{\text{d}}_{ij}z_{ij}}{Q_{i}^{r}}\forall i\in I,k\in K,r=1,2. \end{array}$$

Using a genetic algorithm to solve the model,

(1) Coding scheme
  (13) $$\begin{array}{c} Z:z_{1}z_{2}\text{…}z_{j}. \end{array}$$
(2) Initialization [23]: all facilities must meet the conditions, and the algebraic times at this time *t*=0:
  (14) $$\begin{array}{c} \sum_{j\in J}x_{j}\leq P. \end{array}$$
(3) Fitness function: convert the target value to obtain the individual fitness value:
  (15) $$\begin{array}{c} F\left(i\right)=\frac{1}{f\left(i\right)}. \end{array}$$
(4) Select the operation: until the set special termination conditions are reached, the solution of the model is ended.
  (16) $$\begin{array}{c} p_{i}=\frac{F\left(i\right)}{\underset{N}{\sum }F\left(i\right)}, \end{array}$$
  (17) $$\begin{array}{c} \sum_{i=1}^{i=N}p_{i}<u_{i}\leq \sum_{i=1}^{i=N+1}p_{i}, \end{array}$$
where *i* = 1, 2,…, *N*; *j* denotes the *j*-th alternative address; *J* is the number of selected facilities; *z*_*j*_ is 0 to indicate that it is not selected; and *Q*_*i*_^2^ is 1 to indicate that it is selected. A indicates that the demand point is serviced by 2 implementation points. The model in this article has *P* capacity constraints. *I* ∗ *J* is the nonnegative constraints; *I* ∗ *J* ∗ *K* is the requirement constraints; and *L* represents the sum of the distances from the facility to the grid demand point.

### 2.4. GIS Technology

The GIS auxiliary system carries on the facility location work. It can accurately find the emergency location in urgent need of support and make a series of planning and decision according to the route.

**Figure 2:**
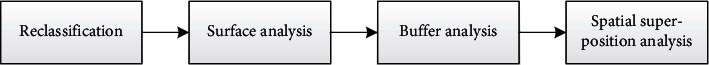
Spatial analysis diagram.

GIS [24], geoscience information system, is a kind of spatial information system that collects, manages, and describes the natural geographic location data. It integrates visual effects and geographical analysis functions of maps, and also adds database operation. In recent years, with the development of various computer technologies, data analysis is becoming more and simpler. With the support of computer technology, it is very important for many applications. It is widely used for smart city construction, transportation system construction, Internet of Things technology, edge computing, and other technologies. Taking ArcGIS as an example, it has a wide range and rich functional modules, and can easily obtain diversified data analysis. Spatial analysis of vector data is shown in Figure 2.

Buffers can be defined as

(18) $$\begin{array}{c} P=\left\{x|d\left(x,A\right)\leq r\right\}. \end{array}$$

The radius of curvature of Maoxi circle:

(19) $$\begin{array}{c} N=\frac{a}{\sqrt{1-e^{2}\sin^{2}\;B}}. \end{array}$$

### 2.5. Load Balancing

#### 2.5.1. Load Information Collection

CPU information: calculate the utilization rate of the CPU and record the information activity time of each CPU.

(20) $$\begin{array}{c} {\text{CPU}}_{\text{use}}=\frac{\left(\text{total}2-\text{total}1\right)-\left(\text{idle}2-\text{idle}1\right)}{\text{total}2-\text{total}1}\times 100\%. \end{array}$$

Load information [25]:

(21) $$\begin{array}{c} {\text{MEM}}_{\text{available}}=\left({\text{Meml}}_{\text{free}}+\text{Buffers}+\text{cached}\right), \end{array}$$

(22) $$\begin{array}{c} {\text{MEM}}_{\text{use}}=\frac{{\text{MEM}}_{\text{total}}-{\text{MEM}}_{\text{available}}}{{\text{MEM}}_{\text{total}}}\times 100\%. \end{array}$$

#### 2.5.2. Load Information Processing

Specific gravity coefficient of CPU of *i* th server node:

(23) $$\begin{array}{c} C_{a}=\frac{C_{i}}{\sum_{i=1}^{n}C_{i}}. \end{array}$$

Specific gravity coefficient of memory:

(24) $$\begin{array}{c} M_{a}=\frac{M_{i}}{\sum_{i=1}^{n}M_{i}}. \end{array}$$

Specific gravity coefficient of disk I/O:

(25) $$\begin{array}{c} D_{a}=\frac{D_{i}}{\sum_{i=1}^{n}D_{i}}. \end{array}$$

Specific gravity coefficient of network bandwidth:

(26) $$\begin{array}{c} W_{a}=\frac{W_{i}}{\sum_{i=1}^{n}W_{i}}. \end{array}$$

Finally, the initial weight value of the server node can be obtained from this formula

(27) $$\begin{array}{c} {\text{weight}}_{\text{init}}=K_{c}\times C_{a}+K_{m}\times M_{a}+K_{d}\times D_{a}+K_{w}\times W_{a}. \end{array}$$

#### 2.5.3. Weight Modification

Average response time of cluster system to process network requests:

(28) $$\begin{array}{c} T_{\text{avg}}=\frac{\sum_{i=1}^{n}T_{i}}{n}. \end{array}$$

Variance of cluster system:

(29) $$\begin{array}{c} D_{t}=\sum_{i=1}^{n}\frac{{\left(T_{i}-T_{avg}\right)}^{2}}{n}. \end{array}$$

## 3. Public Health Medical Education System

### 3.1. Design Principle

The design of this system can effectively manage all kinds of resources and information related to public health education. Our goal is to turn the system into an efficient, convenient, and easy to operate practical tool. While monitoring and detecting public emergencies, effective intervention is carried out through an early warning mechanism. It provides a reliable basis for the decision-making of the government and leaders.

1. Advanced and scalable: on the premise of meeting all functions, consider the update iteration problem and ensure the continuous innovation of data storage and management, which requires scalable. Maintain the advanced technology for a long time in the future and hope to keep pace with the times and ensure the upgradability of software.
2. Practicality: always remember that the ultimate goal of the system is to serve people's needs. According to the specific actual needs, strive to achieve the humanized design. It should not only be simple and easy to operate, but also have a simple and beautiful interface. Whether it is a system upgrade or daily maintenance, it should not be too difficult, but easy to get started.
3. Security: the information and data in the system are confidential and cannot be leaked. Therefore, there should be perfect data backup, authority management, automatic recording of access, monitoring, and other security measures.
4. Openness: support a variety of hardware devices; adopt a standard interface; ensure that the system designed in this article can exchange and share data with other systems; and support secondary development and utilization.
5. Overall situation and integrity: our system is special and applied to emergency handling. Therefore, we must consider the architecture design of the system and the functional design of various modules from a global, multi-faceted, and multi-angle perspective. Ensure that the design is not repeated, the functions are not messy, and there is no unnecessary intersection. This concept can better help us to design the system.

### 3.2. Functional Requirements

According to the actual situation, the functions of our system are as follows:

1. emergency preparedness and construction of emergency resource database
2. improve the early warning mechanism
3. monitoring and testing public health education and medical emergencies
4. emergency treatment and professional services
5. termination and evaluation function
6. construct a data warehouse and manage all kinds of information
7. comprehensive inquiry platform
8. statistical analysis of public knowledge base, case base, database, document report, and other documents as a certain reference
9. site selection of emergency rescue facilities.

Four subsystems are planned to be designed as follows:

1. mobile emergency system
2. Internet of Things peripheral device system
3. GIS monitoring system
4. Public opinion monitoring system

### 3.3. System Design

**Figure 3:**
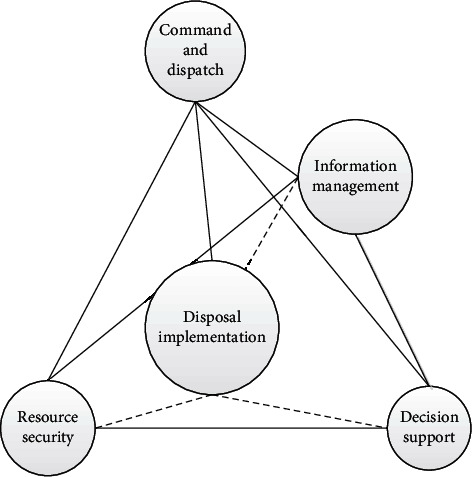
Emergency management diagram.

Generally speaking, the emergency management system has five functional modules. Their respective system functions and responsibilities are different. Among them, command and dispatch are the highest decision-making function and the “total” processing brain is the system. The command system is the most basic and important setting in the whole system construction. It can effectively guarantee normal function. The other four functions are subfunctions, which belong to the support system. They all listen to the deployment of the “brain.” Several functions coordinate and support each other (Figure 3).

### 3.4. Architecture Design

**Figure 4:**
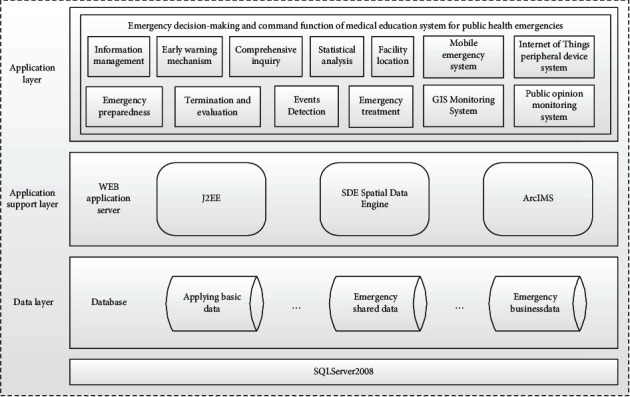
System logical architecture diagram.

The architectural design of the whole system is divided into three parts. The first part is the data layer. The second part is the application of the support layer; rely on the WEB application server in this layer, including J2EE, SDE spatial data engine, and ArcIMS. The third part is the application layer, the practical application of nine functions and four subsystems is realized (Figure 4).

## 4. Experimental Analysis

### 4.1. Development Environment

In this section, we will explain the software and hardware development of the system used in some of the platforms and other related equipment (Table 3).

### 4.2. System Test

Among the evaluation criteria of a system, the solution to the problem of increasing concurrent visits is undoubtedly a great test for the carrying capacity of the system. Once the system is enabled, if our system cannot often face the pressure of a large number of concurrent requests in a short time and the background server cannot bear the huge access pressure, then the system will crash and cannot provide normal services. Through load balancing algorithm and practical application scenarios, the request processing performance of the system is optimized and improved to be more practical, which is more in line with the requirements of this system. To better illustrate the superiority of this method, CoAP, CoCoA, and CoAP-E (i.e., the Internet of Things method introduced in this article) are used to compare the performance and verify the specific situation of scene use under the Internet of Things technology.

#### 4.2.1. Interface Testing

**Figure 5:**
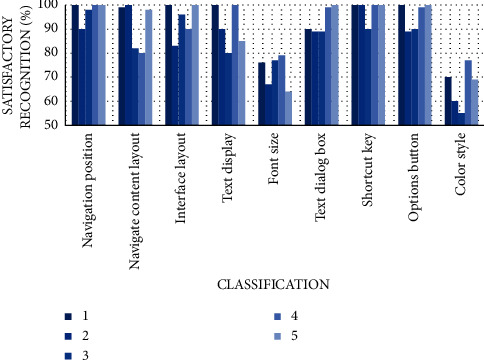
User interface testing.

For the design of a system, we consider the principle of practicality. The user interface we initially finalized needs to be evaluated for external esthetics and internal operation friendliness. To verify the fairness and accuracy of the experiment, voluntary participants were invited and divided the number into five groups. These participants often participate in public emergency rescue and are volunteers who often need to use this system. The number of people in each group for the interface test is controlled at about 10, with a total of 50 people (Figure 5).

According to the results, we observe that everyone has different preferences for the interface and has strong personal subjectivity. To better adjust the public's preferences, the adjustment of the system mainly focuses on the test items with extreme scores. If the scores of other items are determined to be above 80% (including 80%), they will be retained and judged as qualified interfaces. Finally, the parts that need to be adjusted in the system interface test are font size and color style. On the whole, although the interface test basically meets the needs of users, there are still details to be optimized.

#### 4.2.2. Throughput Test

**Figure 6:**
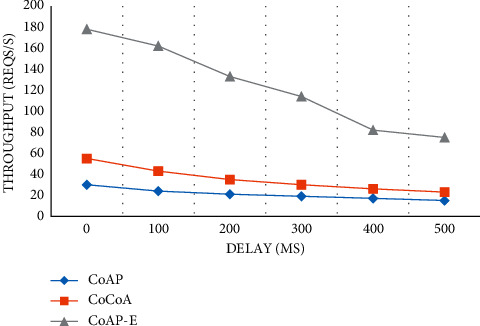
Throughput testing.

This is a test point to prove the superior performance of the system. We can find that with the increasing network delay, the throughput of the three methods also decreases. When the delay is 0, the throughput of the CoAP method is much less than that of other methods. On the whole, the throughput of CoAP-E in this article is obviously superior to the other two methods, which opens a big gap (Figure 6).

#### 4.2.3. Request Success Rate

**Figure 7:**
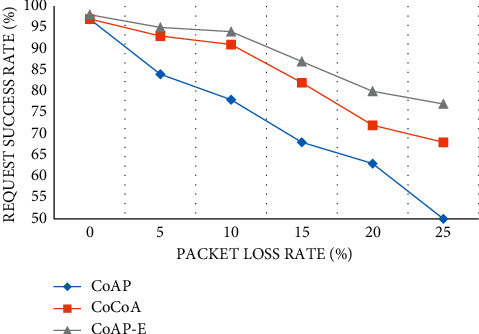
Request success rate.

In this section, according to the change in packet loss rate, the request success rate of the three methods is compared. This test can well reflect the reliability of data transmission. We can find that with the gradual increase of packet loss rate, the request success rate under the three methods is reduced to varying degrees. Among them, the request success rate of the CoAP method shows a “drop” decline. The CoAP-E method has the best performance, followed by the CoCoA method (Figure 7).

#### 4.2.4. Data Retransmission Rate

**Figure 8:**
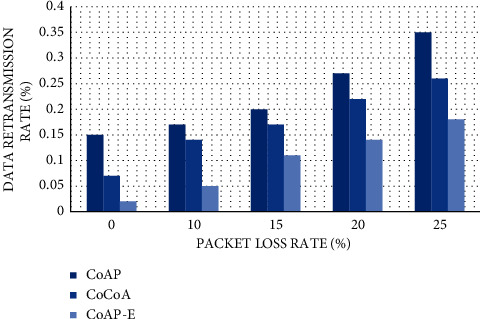
Data retransmission rate.

Reuploading data will cause a waste of resources or excessive overhead for users and clients. Therefore, the smaller the retransmission rate, the better the equipment performance of the system. The test is carried out under the change of packet loss rate. The retransmission rate of this method cannot exceed 18% at most under the packet loss rate of 25%. Under the same packet loss rate, the CoAP method has a data retransmission rate as high as 35%. It is enough to show that the network adaptability of this method is the strongest, so the retransmission rate is the lowest (Figure 8).

#### 4.2.5. Average Response Time

**Figure 9:**
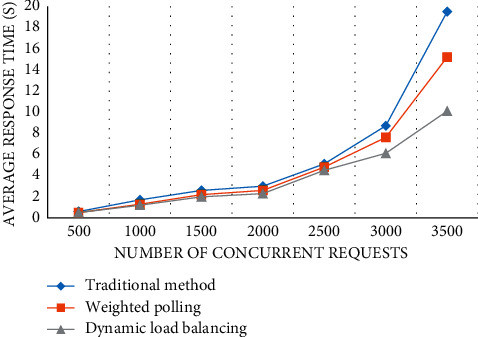
Average response time of the system.

When the number of concurrent requests is less than 2500, the time increase of the three methods is slow, the difference is not big, and the performance distinction is not obvious. However, when the number of concurrent requests reaches 3500, the corresponding time of the traditional method is as high as 19.5 s, and the effect is the worst. The response time of the weighted polling method is about 15.2 s, dynamic load balancing has the best performance, and the duration is 10.1 s (Figure 9).

#### 4.2.6. Site Selection Test

**Figure 10:**
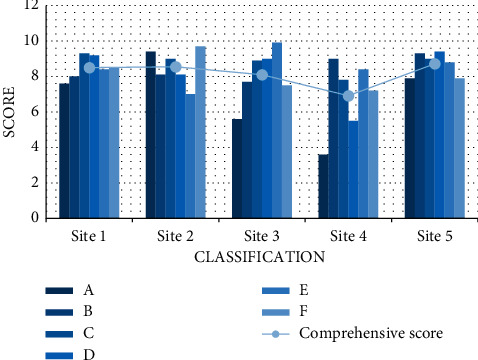
Location score test.

The system selects five sites to evaluate six indexes: A, B, C, D, E, and F. Take a 10-point system to score. Site 4 has the lowest comprehensive score, with a score of about 6.9 points. The scores of Site 1 and Site 2 are about 8.5 points; Site 3 scored 8.1. Only the final score of Site 5 is as high as 8.7 points. Considering all factors comprehensively, the fifth site should be selected as theresettlement site of emergency rescue facilities (Figure 10).

## 5. Conclusion

The rational application of Internet of Things technology can bring excellent performance to the basic construction of the system. According to the requirements of the system, the functional modules are designed. Using GIS technology to quickly respond and select the address of event assistance; through load balancing technology, the concurrency optimization research of Internet of Things technology is completed. Reduce unnecessary system development expenditure and system development time as much as possible. Using computer technology to improve the processing efficiency of the system. According to the experiment and system test, the final research results of this article show that the standard system mode is used to realize the emergency detection and early warning design of public emergencies. The old architecture has been transformed, and new technologies such as the Internet of Things has been added. It mainly aims at public health emergencies and solves the problems in medical care and education. The system can accumulate and optimize the experience and methods to solve emergency incidents. The system can effectively improve the efficiency of the government and staff to deal with events, and coordinate the relevant interests of the public and the people. However, there are still many problems and deficiencies in this system, which need to be continuously optimized and improved. For example, the requirements analysis of the system should also be further excavated and studied to understand the specific relationship between problems and functions. The system needs a more humanized design, close to people's lives, and professionals only need simple training to get started. The performance optimization of the Internet of Things system needs to be tested according to the actual use to obtain more accurate and true performance. In this experiment, the selection range of some variables and parameters is narrow and the analysis is limited, and the problems with a wider range and more requirements need further study; For future research, GIS technology and Internet of Things technology should be better combined, and Internet big data should be dynamically used so that the target selection can get the optimal solution. These are the problems that must be further faced and solved in the research process.

## Figures and Tables

**Table 1:** Definition of event characteristics.

Type	Characteristics of an event	Notes and remarks
Public emergencies [18]	The characteristic of being sudden, usually occurring in an explosive manner on a large scale	An emergency may be a public emergency. But it may not be included relationships [19].
An emergency	A more urgent event. Usually, the time for processing is very tight	
Hazardous events [20]	Such incidents have usually produced dangerous and serious consequences	The biggest difference between the two types of events is that crisis is a hazard that has not yet happened. It is potential, but it shows serious consequences.
Crisis event	This is a serious state and there is a potential danger	
Disaster event	Events usually caused by natural factors, which are very destructive	Disaster events can be regarded as a higher level escalation of disaster events, with higher severity and greater destructiveness. Catastrophic events may happen suddenly, but they may also be expected.
Catastrophic events [21]	Destructive, serious	

**Table 2:** Classification and grading of events.

Classification of events	Classification of events
A major epidemic of infectious diseases	Particularly important events are set to level I
Group diseases of unknown origin [22]	Major events are set to level II
Major food and occupational poisoning	Larger events are set to level III
Other events seriously affecting public health	General events are set to level IV

**Table 3:** Development environment of the system.

Environment	Name	Specific equipment
Software environment	System software	Microsoft Windows 2000/XP
	Database (DBMS)	IBM DB2
	Application server	IBM WebSphere
Hardware environment	Database server	The main frequency requires a dual CPU with the main frequency above 2.0 GHz; memory requirements of more than 1 GB; the hard disk requires more than 80 GB.
	Web server	The main frequency is required to be above 2.0 GHz, and the main frequency is double CPU; memory requirements of more than 1 GB; the hard disk requires more than 60 GB.
	Map server	The main frequency requires a dual CPU with the main frequency above 2.0 GHz; memory requirements of more than 1 GB; the hard disk requires more than 40 GB; the network card requires a network adapter card above 100M; the display requires VGA or super VGA display with resolution above 1024∗768; the graphics card requires a display card with more than 64M video memory.

## Data Availability

The data used to support the findings of this study are available from the corresponding author upon request.
